# Predicting the Responses of Functional Leaf Traits to Global Warming: An *In Situ* Temperature Manipulation Design Using *Iris pumila* L.

**DOI:** 10.3390/plants12173114

**Published:** 2023-08-30

**Authors:** Sanja Manitašević Jovanović, Katarina Hočevar, Ana Vuleta, Branka Tucić

**Affiliations:** 1 Department of Evolutionary Biology, Institute for Biological Research “Siniša Stanković”—National Institute of the Republic of Serbia, University of Belgrade, 11108 Belgrade, Serbia; katarina.hocevar@ibiss.bg.ac.rs (K.H.); ana.vuleta@ibiss.bg.ac.rs (A.V.); 2 Independent Researcher, 11070 Belgrade, Serbia; btucic@sbb.rs

**Keywords:** global warming, open-top chamber, functional leaf traits, SLA, LDMC, SLWC, LT, *Iris pumila* L.

## Abstract

Phenotypic plasticity is widely acknowledged as one of the most common solutions for coping with novel environmental conditions following climate change. However, it is less known whether the current amounts of trait plasticity, which is sufficient for matching with the contemporary climate, will be adequate when global temperatures exceed historical levels. We addressed this issue by exploring the responses of functional and structural leaf traits in *Iris pumila* clonal individuals to experimentally increased temperatures (~1.5 °C) using an open top chamber (OTC) design. We determined the phenotypic values of the specific leaf area, leaf dry matter content, specific leaf water content, and leaf thickness in the leaves sampled from the same clone inside and outside of the OTC deployed on it, over seasons and years within two natural populations. We analyzed the data using a repeated multivariate analysis of variance, which primarily focusses on the profiles (reaction norms (RNs)) of a variable gathered from the same individual at several different time points. We found that the mean RNs of all analyzed traits were parallel regardless of experienced temperatures, but differed in the level and the shape. The populations RNs were similar as well. As the amount of plasticity in the analyzed leaf trait was adequate for coping with elevated temperatures inside the OTCs, we predict that it will be also sufficient for responding to increased temperatures if they exceed the 1.5 °C target.

## 1. Introduction

The current century has witnessed climate warming on an unprecedented scale. The Earth’s global surface temperature in 2022 was 0.89 °C warmer than the twentieth century average, and 1.09 °C warmer than in 1880, when modern recordkeeping began [1,2,3]. There is a general consensus that increasing emissions of greenhouse gases from human activities, notably carbon dioxide and methane, are causing a rapid global surface temperature increase worldwide [1,2,3]. This small, but significant, long-term rise in the accumulated heat has altered a wide range of climate variables (e.g., weather and climate extremes) in every region of the Earth [1,2,3]. The latest climate models predict that, without a sharp decline in greenhouse gas emissions by 2030, global warming will surpass 1.5 °C in the following decades, threatening extinction for a large number of species [4,5,6].

Anthropogenic climate warming has altered environmental conditions that plants experienced in their recent evolutionary history [7]. Given that the Earth’s atmosphere is heating up, one of the most pressing questions in the studies of plant adaptation to climate warming is how plant populations will respond to ongoing changes in habitat conditions [8,9,10,11,12,13,14]. Theoretically, the process of adaptation to new environmental conditions in the original habitat potentially occurs through two mechanisms: phenotypic plasticity [15], the ability of single genotypes to produce the phenotypes that match the climate they encounter [7,10,16,17,18,19], and genetic evolution, the process by which individuals with genes determining traits suitable for the new environmental conditions increase in frequency over generations [20,21,22,23,24]. In spite of growing evidence on the eco-evolutionary consequences of climate change, our understanding of how phenotypic plasticity and genetic evolution (inter)act to facilitate population persistence following climate warming is still limited [4,9,10,11,12,24].

Phenotypic plasticity is frequently assumed to be an important mechanism by which plants can cope with novel environmental conditions such as those caused by global warming [8,11,25,26]. Phenotypic plasticity allows a population to respond quickly to environmental variation by producing better-matching phenotypes within a single generation. Hence, plasticity would be “a first stage of developmental rescue, prior to subsequent adaptation and evolutionary rescue of a population” [27]. Using individual-based simulation, Scheiner et al. [10] recently found that the induction of phenotypic responses that move trait values (even partly) towards a new adaptive peak in a linearly changing environment can potentially keep pace with rapid environmental changes, thus averting population extinction. However, in an environment with accelerating change, the magnitude of plasticity needs to be much greater or the existing standing genetic variation must be much higher to prevent extinction risk relative to linear environmental change with the same mean rate of change [12]. Using digital organisms, that is, self-replicating computer programs, Lalejini et al. [28] found that adaptive plasticity allows a plastic population to efficiently preserve more new adaptive traits in fluctuating environments compared with their non-plastic counterparts.

Both intra-annual (seasonal) and inter-annual climatic variation can promote increased phenotypic plasticity in plants [29,30,31]. While seasonal variation can select greater plastic responses in reversible physiological traits, inter-annual climatic variation can drive higher plasticity in plant size and allocation [17,32]. The results of a meta-analysis of global data on phenotypic plasticity have indicated that phenotypic plasticity for plant allocation was positively associated with climatic variation, whereas the plasticity of other plant traits, including leaf morphology, physiology, and size, was positively correlated with the average annual temperature [13].

One of the research questions that are still unresolved, and has no scientifically proved answer, is whether the existing level of plasticity in natural populations that appear to be sufficient for coping with contemporary temperature conditions will be enough once global surface temperature exceed the historical level. Given that the climate of a region defines different elements, including solar radiation, temperature, humidity, precipitation, atmospheric pressure, and wind, the answer to the posed question requires the use of specific experimental approaches that allow for separating the effect of air temperature on individual plants and plant populations from the effects of other climatic elements, if possible, in nature [11].

**Figure 1:**
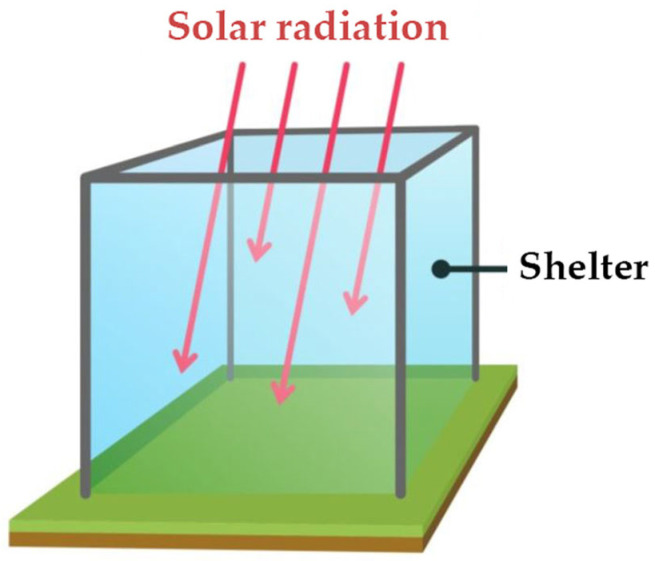
The scheme of an open top chamber (OTC) used in this study.

Among the different techniques that have been applied for modulating air temperature in the field, *in situ* passive experimental warming by means of open top chamber (OTC) devices (Figure 1) appears to be very appropriate [33,34,35], especially for disentangling the “cause-and-effect” relationships between plant responses and climate change [36]. Passive warming chambers have been widely applied to assess the impacts of climate warming on plant communities in Alpine, Arctic, and Antarctic regions [33,34,37,38,39,40], as well as in the Tibetan Plateau [41,42,43]. According to Elmendorf et al. [40], open-top designs for manipulating field temperature “yield consistent estimates of the magnitude of response of plant communities to climate warming”, and as such are “best suited for forecasting impacts over the coming decades”.

Our overarching objective in this study was to assess whether plastic phenotypic responses in leaf functional traits induced by the mean daily temperature increase of ~1.5 °C *in situ*—the global climate target—provide a potential mechanism by which plants can match quickly to novel environmental conditions (i.e., plasticity rescue) [25] prior to subsequent adaptation (i.e., evolutionary rescue) [44]. To do so, we carried out a temperature manipulation experiment by OTCs in nature, using clonal individuals of a perennial monocot, *Iris pumila* L., which inhabits exposed dune sites in the Deliblato Sands, Serbia. Here, we analyzed intra-annual (seasonal) and inter-annual variation in the phenotypic expressions of four key leaf traits: specific leaf area (SLA), leaf dry matter content (LDMC), specific leaf water content (SLWC), and leaf thickness (LT) in leaves developed on the same clonal plants inside and outside of every OTC.

The leaf traits analyzed in this study are categorized as “functional traits” as they reflect various aspects of resource uptake and utilization [45]. Specific leaf area (SLA) (area/dry mass) is the major determinant of the plant resource capture strategy [46]. SLA depends strongly on irradiation level [47], water supply [48] and nutrient availability [49,50]. Leaf dry matter content (LDMC) and leaf thickness (LT) are indicators of resource protection and conservation strategy [51,52]. LDMC is positively correlated with leaf life-span and negatively correlated with relative growth rate [53]. Leaf thickness (LT) affects the quantity of absorbed light and the speed of carbon dioxide diffusion [54]. LT is negatively associated with the rates of photosynthesis [55] and growth [56,57]. Because of this, LT has often been used as an indicator of ecological plant performance [58]. Specific leaf water content (SLWC) is commonly used as a predictor of plant whole-leaf photosynthesis and leaf area [59]. SLWC correlates well with nitrogen concentration and assimilation capacity [51], as well as with leaf tissue density and thickness [60].

An important prerequisite for making predictions about the adaptiveness of the phenotypes expressed in response to global warming is to characterize the shape of their reaction norms [61,62], i.e., the function that describes the dependence on the environmental conditions of phenotypes produced by the same genotypes [63,64]. Assessment of variation in reaction norms is of vital importance for understanding how natural populations will respond to ongoing climate change, as well as for predicting how non-genetic plastic responses can avert populations from extinction in a warming world.

The primary goal of this study was to compare plastic responses (reaction norms) of the four functional leaf traits—SLA, LDMC, SLWC and LT—expressed by the same clonal genotypes of *Iris pumila* under natural and experimentally elevated air temperatures in the field. We addressed the following questions: (1) What is the mean shape of plasticity (i.e., the species-specific reaction norm) in response to the growth temperature in *I. pumila*? (2) Does the magnitude and direction of the mean plastic responses to the growth temperature vary among leaf traits seasonally and/or annually? (3) Are the population-level reaction norms to variation in the growth temperature leaf-trait-specific?

## 2. Results

### 2.1. Abiotic Environmental Conditions

The climatic parameters, which illustrate clonal micro-environmental conditions prevailing within sampling sites, are given in Table 1. The measurements of instantaneous air and soil temperature, as well as soil moisture, were taken just before leaf sampling. The mean instantaneous air and soil temperatures differed between the alternative temperature environments. The mean air temperature was higher inside the OTCs, while the corresponding soil mean temperature was lower compared with that prevailing outside of the OTCs. A reversed trend was observed outside the OTCs: the mean air temperature was lower, but the mean soil temperature was higher relative to that recorded inside the OTCs. Both *I. pumila* populations exhibited similar temperature trends over the entire experimental period, i.e., throughout the two seasons, spring and summer, and two consecutive years, 2018 and 2019. In contrast with that, the mean soil moisture (expressed in percentage) appeared to be relatively constant over all of the clonal individuals, regardless of the ambient temperature they experienced. The seasonal variation in the air temperatures, both inside and outside the OTCs showed a contrasting trend between the two successive years. In 2018, the mean temperatures had greater values in the spring compared with the summer. However, in 2019, the summer mean air temperatures were greater than the spring air temperature means. Soil moisture showed the opposite trend; the mean soil moisture in spring 2018 was lower than in spring 2019, whereas in summer 2019, the mean soil moisture was higher relative to that recorded in summer 2018. Considering the mean soil temperature, in both years, the summer mean values were constantly higher than those recorded in the spring. Thus, the climate conditions prevailing in Deliblato Sands during 2018 and 2019 characterized an exceptionally dry and hot spring and rainy summer in 2018, followed by a drizzly spring and hot, dry summer in 2019. Similar to instantaneous air temperatures, the mean air temperatures recorded by data loggers were consistently higher inside the OTCs compared with the natural ones prevailing outside the OTCs (Table 1). 

### 2.2. Phenotypic Responses of SLA, LDMC, SLWC, and LT to Temperature

**Figure 2:**
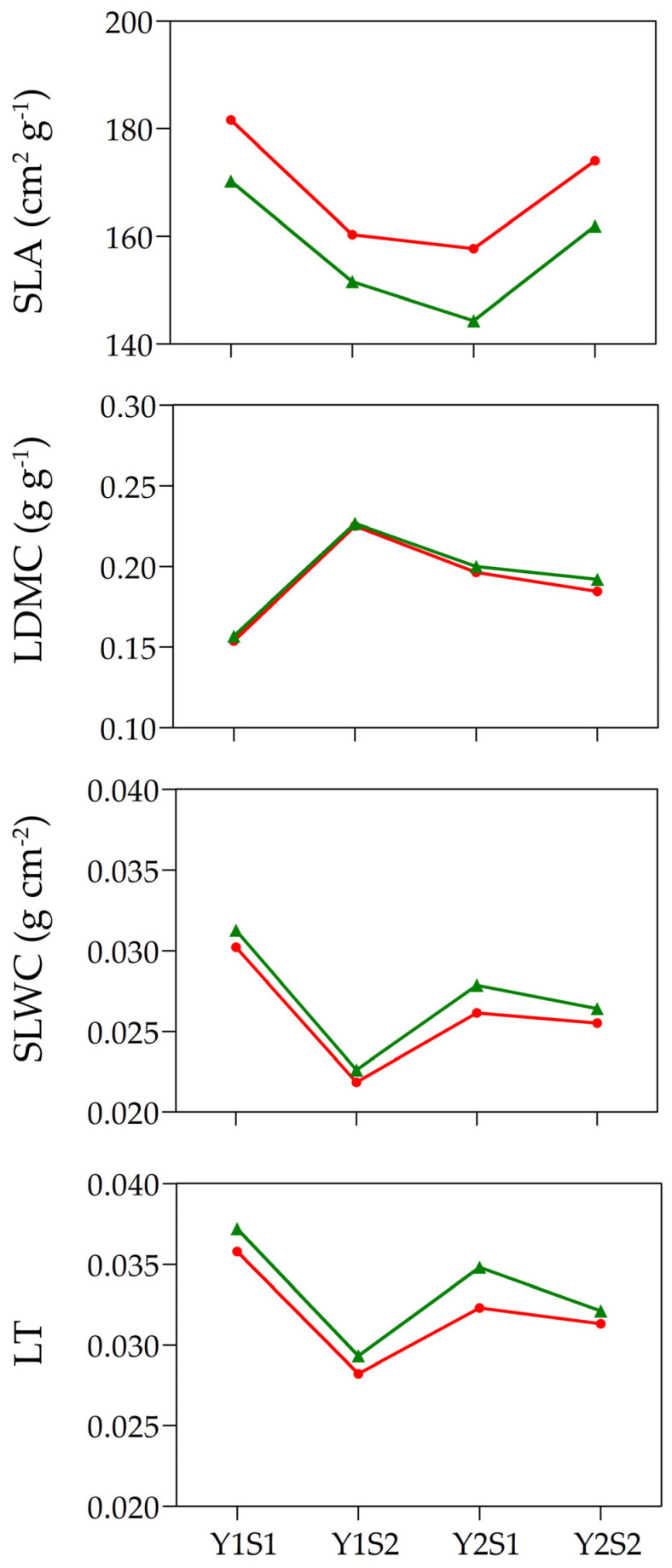
Plots of temporal reaction norms for the mean values of the specific leaf area (SLA), specific leaf water content (SLWC), leaf dry matter content (LDMC), and leaf thickness (LT) of *Iris pumila* clonal individuals from two natural populations (Population 1, n = 24 and Population 2, n = 21), enduring from the spring 2018 (Y1S1), over summer 2018 (Y1S2), and spring 2019 (Y2S1) to the summer 2019 (Y2S2), as expressed outside (green line, triangle symbol) and inside (red line, circle symbol) of the OTCs in the Deliblato Sands. Y = year, S = season.

To examine the phenotypic responses of the leaf functional traits to temperature, the values of SLA, LDMC, SLWC, and LT were measured over time on the same clonal individuals of *I. pumila* occupying two sun-exposed natural populations. The leaves were sampled inside and outside the OTCs once for each season (spring and summer), during a two-year period. The mean values and corresponding standard errors for SLA, LDMC, SLWC, and LT are presented in Table 2. As can be seen, the phenotypic values of all of the examined functional leaf traits changed with air temperature. In both populations, throughout the whole vegetation season, the mean SLA values were higher (up to 9%) in the ramets developed inside an OTC, at an experimentally elevated temperature, compared with that developed outside the same OTC. An inverse pattern was seen for LDMC, SLWC, and LT, which appeared to be lower in the leaves experiencing elevated air temperatures than those grown under ambient temperature conditions. All of the changes described were similar during both experimental years (Figure 2, Table 2 and Table 3). 

### 2.3. Reaction Norm Graphs for SLA, LDMC, SLWC, and LT

The mean reaction norms of SLA, LDMC, SLWC, and LT for leaves of *I. pumila* developed inside and outside the OTCs are depicted in Figure 2. The reaction norms of most leaf traits were parallel over the whole research interval. The only exception was LT, which had parallel reaction norms between the time points Y1S1 and Y1S2, and convergent ones at the time point Y2S2 (Figure 2). The level of reaction norms was also trait-specific. The mean reaction norms of SLA ranked higher at elevated temperatures (inside the OTCs) than at an ambient temperature, in contrast with LDMC, SLWC, and LT, which had greater levels outside the OTCs than inside of them (Figure 2). The variation in the shape and the level of reaction norms appeared to be trait- and time-interval specific (Figure 2). For example, the levels of reaction norms for SLA, SLWC, and LT were decreasing between spring and summer of the first year (time points Y1S1 and Y1S2), but increasing in the second year (time points Y2S1 and Y2S2) for SLA, and decreasing for SLWC and LT. LDMC was the only leaf trait for which reaction norms exhibited increasing levels between time points Y1S1 and Y1S2, but a slightly decreasing levels between time points Y2S1 and Y2S2. Interestingly, the shape of reaction norms over time changed from concave for SLA to convex for LDMC to zig-zag for SLWC and LT at both temperature regimes (Figure 2).

### 2.4. Profile Analysis

To examine whether there is a significant difference between the mean response profiles of plants grown under different temperature conditions, we applied a profile analysis on functional leaf traits: SLA, LDMC, LWC, and LT. The results of the profile analyses are presented in Table 3. Because this procedure consists of three tests—the test of parallelism, the test of level, and the test of flatness—and as these tests should be carried out in a sequential order, we started by testing whether the mean response curves were parallel. In terms of the profile analysis, we tested whether there was a significant interaction between the within-subject main effects (year, season) and the temperature treatment. The profile analysis revealed that the probabilities of F-statistics (*p* > F) for all of the interaction effects (treatment-by-season, treatment-by-year, and treatment-by-year-by-season) were statistically insignificant at α = 0.05 (Table 3). A lack of significant interactions between the within-subject main effects and the temperature treatment corroborated the null hypothesis that the mean response profiles of SLA, LDMC, SLWC, and LT between plants grown inside the OTCs and plants grown outside the OTCs were parallel over time.

Given that the mean response profiles of the two groups were parallel for each trait being studied, we tested whether the profiles were at equal levels (i.e., there was no group difference). The profile analysis revealed that the treatment effect for all four leaf traits was statistically significant (SLA, *p* < 0.0001; LDMC, *p* = 0.0058; SLWC, *p* = 0.0007; and LT, *p* = 0.0002; Table 3), indicating that the levels of the two groups were significantly different from one another. Based on this test, the hypothesis of equal levels was rejected. 

The profile analysis of the between-population effects showed that the mean response profiles between populations for all of the analyzed traits were the same for SLA and LDMC (all *p*s > 0.05), but differed for SLWC and LT (*p* = 0.0063 and *p* = 0.0092, respectively). Regarding SLA and LDMC, the results indicated that the plants from both populations responded similarly to elevated temperatures. 

The tests of flatness hypotheses are presented in Table 3. The statistically significant multivariate *F*-values for the season and year main effects suggested that functional leaf traits elicited the same average response to temperature variation: SLWC and LT over the years (*p* = 0.7338 and *p* = 0.9927, respectively) and SLA over the seasons (*p* = 0.1558). All of the traits changed their response profiles either over the seasons (LDMC, SLWC and LT; all *p*s < 0.0001) or across the years (SLA and LDMC; *p* = < 0.0001, *p* = 0.0474, respectively).

Individual ANOVAs (F-tests) on each of the contrasts for the main within-subject factors, namely season and year, revealed that, when averaged over both treatments, there was a significant change in all of the analyzed leaf traits over time (a significant mean effect for all ANOVAs; Table 4). A highly significant treatment effect (*p* = 0.0001) was obtained exclusively for LT, indicating that the mean response profile between the two groups differed only in the second year of repeated measurements (Table 4). 

Overall, our results suggested that the response profiles of functional leaf traits in clonal individuals of *I. pumila* growing inside and outside OTCs were parallel, but not coincidental and flat. This implies that an increase in air temperatures of about 1.5 °C can evoke changes in both the level and shape of the mean response profiles of leaf traits in *I. pumila* compared with those expressed at ambient air temperatures.

### 2.5. Kendall Rank Correlations between Functional Leaf Traits

The results of the Kendall rank correlation analyses revealed that functional leaf traits in *I. pumila* were mostly negatively correlated with one another, with the exception of LT, which was positively correlated exclusively with SLWC in both of the temperature treatments. The correlation between SLWC and LT was the highest compared with all of the other correlations (Tau b = 0.8673 and Tau b = 0.8834; both *p* < 0.0001, in the high and low temperature treatments, respectively) between the functional leaf traits. Correlations between LT and LDMC were negative in sign and moderate in strength (Tau b = −0.559 and Tau b = −0.502, both *p* < 0.0001, in the high and low temperature treatments, respectively), as were the correlations between SLWC and LDMC (Tau b = −0.623 and Tau b = −0.589, both *p* < 0.0001, in the high and low temperature treatments, respectively). The lowest negative correlations were detected between SLA and LDMC (Tau b = −0.389 and Tau b = −0.353, both *p* < 0.0001, in the high and low temperature treatments, respectively), and between SLA and LT in the low temperature treatment (Tau b = −0.145, *p* < 0.0039, in the low temperature treatment).

### 2.6. Regression Analysis

To better understand the association between air temperature and leaf functional traits, we employed a robust regression analysis. The results of the robust regression analyses have shown that the relationship between SLA and the mean daily temperature (Logged air temperature, T_L_) was generally non-significant, with the exception of a marginally significant association with T_L_ in the summer (b = 4.2954, *p* < 0.06). LDMC exhibited a significantly positive relationship with T_L_ over the whole experiment (b = 0.0050, *p* < 0.0001). Conversely, the association between T_L_ and both SLWC and LT was significantly negative (for both traits b = −0.0009, *p* < 0.0001).

## 3. Discussion

The goal of this study was to obtain a broader insight into the phenotypic changes of functional leaf traits that may likely occur in response to global climate warming, as well as to assess whether these changes are sufficiently fast to keep pace with rapid shifts in the environment. To achieve this goal, we compared the average reaction norms of the four functional leaf traits, SLA, LDMC, SLWC, and LT, in *I. pumila* clonal individuals. This comparison was made in response to air temperatures experienced inside and outside the OTCs over two successive vegetation seasons. As “a reaction norm is a mirror that reflects environmental effects into phenotypes” [65], and because each clonal individual has simultaneously experienced both the natural and experimentally increased (~1.5 °C) air temperature, we were able to forecast the magnitude and the direction of plastic changes in functional leaf traits that might occur if the global surface temperature approaches the 1.5 °C global goal.

Very recently, Arnold et al. [62] pointed out that a precursor to a better understanding of how phenotypic plasticity can potentially facilitate population persistence in novel and rapidly changing environments is to characterize the shape of reaction norms. The mean shape of reaction norms for the analyzed leaf traits in *I. pumila* appeared to be markedly dissimilar across the same environmental range, but very alike when compared under natural and experimentally elevated air temperatures. The results of a MANOVAR analysis corroborated the graphical features of these reaction norms. The mean reaction norms of the four analyzed leaf traits expressed at alternative temperature regimes were parallel over time, but not coincidental and flat. According to the obtained results, it seems reasonable to believe that functional leaf traits of *I. pumila* have the capacity to respond plastically to temperature increases due to global change, as well as that the rate of these plastic changes can be very fast, occurring within a single vegetative generation of this clonal plant. However, it is difficult to anticipate whether the plasticity of functional leaf traits to rapid temperature increase (as occurs presently due to global change) will generate phenotypes that match well with novel environmental conditions. Snell-Rood et al. [25] suggested that initial plastic responses to novel environmental conditions are, in many cases, maladaptive due to the absence of past selection in these environments. As microclimatic environmental conditions experienced by individual *I. pumila* clones differed exclusively in air temperature prevailing inside and outside the OTCs and because there was no signature of negative phenotypic selection operating inside the OTCs (e.g., dead ramets), it is likely that the phenotypic changes of leaf functional traits elicited in response to higher air temperatures inside the OTCs can thus be classified as adaptive.

The leaf traits included in this study are components of the leaf economics spectrum (LES) [66], whose variation and covariation reflect an ecological trade-off in the resource acquisition and utilization strategies of plants [46,58,67,68,69]. As such, these traits are expected to be especially affected by global climate change [70,71]. 

One of the most important leaf functional traits is the specific leaf area (SLA). This trait reflects the efficiency of resource utilization strategies of plants [70,72]. It has been recognized that the most influential factor in determining the spatial variation in SLA on a large scale is solar radiation. On a small spatial scale, however, SLA usually increases with the increase in available resources [70]. In our *in situ* temperature manipulation experiments, reaction norms of SLA had a consistently higher level at an elevated temperature than at a natural one, regardless of the season or year. However, when the phenotypic responses of SLA to temperature variation were averaged over both treatments, the reaction norm shape of this leaf trait changed over both seasons and across years. This result is in agreement with the statement that SLA responds nonlinearly to environmental change [70]. SLA was the only leaf trait measured in this study that expressed greater phenotypic values inside rather than outside of the OTCs. This conclusively means that producing leaves with a larger surface area relative to mass can be advantageous in warmer environments because of a greater capacity for light interception, as well as a higher cooling efficiency through transpiration. In spite of numerous pieces of evidence that SLA increases with temperature [73,74,75], the relationship between SLA and temperature is not universally positive, but can vary among plant species, ecosystems, and environmental conditions [75,76]. For example, some studies have provided evidence that SLA tended to decrease in response to elevated temperatures in order to withstand heat stress, either by reducing leaf expansion or increasing leaf thickness [77,78,79].

Leaf dry matter content (LDMC) is a component of LES [66] that is frequently used as an indicator of the position of a given plant species in “a fundamental trade-off between a rapid assimilation and growth at one extreme, and efficient conservation of resources within well-protected tissues at the other” [80] (p. 955). Accordingly, species with faster growth rates will usually exhibit thinner leaves with low LDMC values, fast resource acquisition rates, and leaf turnover, while species with slower growth rates are characterized by thicker leaves with high LDMC values and slower rates of leaf turnover [81,82,83]. However, in the face of rapid climate change, the phenotypic plasticity of LES traits can additionally impact the rate of plant growth [75]. We found that the mean reaction norms of LDMC in *I. pumila* were parallel, but exhibited distinct levels—greater at lower air temperatures—outside the OTCs and lower at higher temperatures—inside the OTCs. In addition, a MANOVAR analysis revealed that the shape of the reaction norm for LDMC changed both seasonally and interannually. The relationship between LDMC and global mean temperature is thought to be complex, as well as context- and species- or ecosystem-dependent [84,85,86]. Because both nutrient uptake and assimilation rates can be amplified at higher temperatures, a tendency for LDMC to decline with temperature increase could be attributed to a greater investment of leaf nitrogen in photosynthetic organs and, consequently, higher leaf respiration and biomass production rates [87]. Warming can indirectly affect LDMC through its direct effect on water content in plant tissue. Specifically, high temperatures can increase evapotranspiration rates, leading to greater water loss from the leaves and, in turn, LDMC decline [88]. In the case of *I. pumila*, the plastic changes in LDMC when subjected to experimentally increased temperatures that simulated the global target of 1.5 °C, altered in a direction that would likely maintain fitness in that microenvironment. Hence, it seems reasonable to believe that the plasticity of LDMC to temperature variation is adaptive, and that its amount is adequate for keeping pace with global warming. 

The specific leaf water content (SLWC) is an important component of LES owing to its key role in leaf thermal regulation and carbon assimilation, as well as its strong correlations with other functional leaf traits [89,90]. The changes in precipitation patterns and water availability associated with global warming can affect the overall water status of plants, influencing SLWC [90]. As different plant species display diverse capacities to adjust to or tolerate changes in temperature and water availability [91], and because temperature and SLWC quantitatively correlate with other leaf traits, such as photosynthetic capacity and SLA (or LDMC), SLWC can be used as an indicator of species-specific adaptation to environmental conditions [90]. In *I. pumila*, the reaction norms of SLWC appeared to be parallel over time at different temperature regimes, but their level was higher at the lower ambient temperature than at the higher one, indicating a negative impact of high air temperatures on leaf water availability. In contrast with the previous reports that variation in LDMC and, thus, in SLWC, are relative constants over seasons [92], our results strongly indicate that the shape of reaction norms for SLWC did change over seasons and across years. The observed temporal changes in the average plastic responses of SLWC in *I. pumila* can be attributed to the immediate alteration in microclimate conditions taking place within their natural habitats. 

Leaf thickness (LT) is a quantitative trait related to leaf form. In the context of the LES, LT reflects a tradeoff between a rapid growth versus drought and heat tolerance. Thinner leaves have a higher surface-to-volume ratio, which enhances the leaf’s ability to dissipate excess heat through increased convective cooling [78,93]. By reducing leaf thickness, plants enhance their heat tolerance and minimize the risk of overheating at high air temperatures [94]. Low leaf thickness indicates a reduced structural support and a potentially limited water storage capacity [95,96]. The reduction in LT can be viewed as an adaptive modification that mitigates heat stress and optimizes heat dissipation [97]. The relationship between LT and warming was found to vary depending on plant species, environmental conditions, and the magnitude and duration of warming [98]. We found that LT in *I. pumila*, tended toward lower values at higher air temperatures compared to its lower counterparts. Similar to other leaf traits measured, the reaction norms of LT were parallel to each other, while their level was greater at lower than at higher air temperatures. The shape of the reaction norms for LT is very similar to that depicted for SLWC, regarding both the intraclonal temperature variation as well as the seasonal and interannual environmental changes. Thus, producing thinner leaves at elevated temperatures would be a rapid phenotypic response that would protect leaf tissue from overheating by increasing the heat dissipation rate owing to a larger leaf surface area.

The phenotypic correlations among the four functional leaf traits, which are components of LES, were similar in strength, irrespective of the temperature treatment, reflecting a conservative plant strategy of *I. pumila* in terms of resource acquisition and allocation. Our results corroborate the view that LES—although present in different plant forms—appeared to be greatly independent of climate conditions.

## 4. Materials and Methods

### 4.1. The Study Species

The dwarf bearded iris, *Iris pumila* L. (Iridaceae), is a rhizomatous perennial herb indigenous to the Eurasian Steppe belt. The species extends from Austria in the west, through central and southeastern Europe, to west Siberia in the east [99,100,101,102,103,104,105,106]. 

*Iris pumila* is a component of the grassland steppe ecosystem (the order Festucetalia valesiacae; [107,108] distributed in the Deliblato Sands (44°90′23″ N, 21°11′32″ E)—a large continental sandy area situated in the southeastern part of the Pannonia Plain, in Banat, Serbia [99,108]. Within that region, natural populations of *I. pumila* mostly inhabit sun-exposed sites on the crests and windward slopes of sand dunes. The species propagates from creeping rhizomes (modified stems) that branch laterally from the central “mother rhizome”, producing first order branches—the “primary segments”. Primary segments continue to branch, resulting in secondary, tertiary, and higher-order branches. The rhizome segments of *I. pumila* are approx. 2.5 cm long and tightly packed, creating round-shaped clones of variable sizes (30–120 cm in diameter), depending on the clone age [109,110]. The blooming phase of *I. pumila* is in early spring. Flowers display a conspicuous within-population color variation. Violet, blue, and yellow color categories are more frequent, but white-colored flowers can be found as well [111]. Flower color variation in *I. pumila* results from the accumulation of multiple pigment classes such as anthocyanins and carotenoids, and their relative content in the petal tissue [112]. Because the biosynthesis pathways of anthocyanins and carotenoids are genetically controlled by structural and regulatory genes [113,114], flower color can serve as a reliable genetic marker for discrimination between district floral genotypes within color-polymorphic populations [111].

### 4.2. Open Top Chambers and Experimental Design

**Figure 3:**
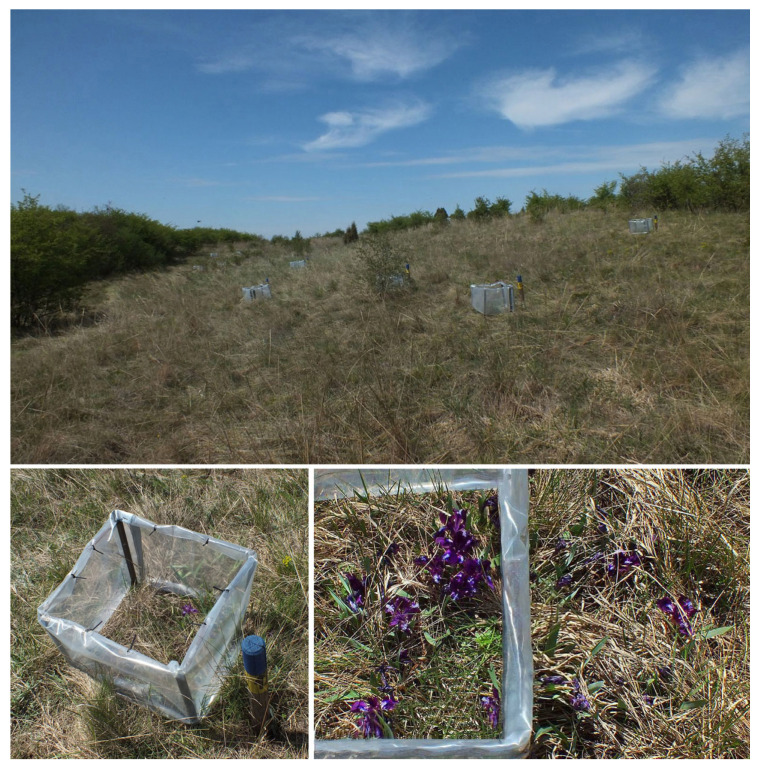
The open top chambers (OTCs) deployed on individual clones of *Iris pumila* naturally growing on a dune slope in the Deliblato Sands (**upper** picture). Perspective and aerial views of the OTC positioned over *I. pumila* clone (**lower** picture).

To simulate global warming, we used a four-sided (50 cm × 50 cm and 50 cm, L, W, H) open top chamber (OTC) design (Figure 3). The walls of the OTCs were made of a diffused light thermal film based on ethylene vinyl acetate (Guarniflon S.p.a., Treviso, Italy), which did not change the spectral composition of the transmitted light, so that the ratio of the red to far-red light (RFR) within the OTCs remained unaffected. The thermal film was attached to four metal pales that were positioned vertically in the corners of the OTC and buried approx. 20 cm in the soil. The chambers passively increased air temperatures at 25 cm height by 1.5 °C above ambient levels, on average, over an entire year.

For this study, we chose two sun-exposed populations of *I. pumila* occurring in the dune system of the Deliblato Sands. One of these populations (hereafter named Population 1) inhabits the slope of a dune, whereas the other population (Population 2) resides along a dune crest (top). The areal distance between these populations is about 4 km.

In April 2017, during the blooming phase of *I. pumila*, we randomly selected a total of 45 large clones (more than 60 cm in diameter), 24 from Population 1 and 21 from Population 2, and marked each of them with a wooden pole displaying clone ID. To protect the intermixing with alien clones after the blooming phase, we removed all flowering ramets differing in coloration from the focal clone that were growing in its vicinity of 30 cm. In May 2017, after the flowering phase ended, we positioned the OTCs over one half of each selected clone, leaving the other half to experience natural environmental conditions (Figure 3). 

### 4.3. Measuring Environmental Variables

Within each of the two populations, we recorded the air temperature inside and outside of the OTCs using a thermal data logger with two external sensors (ThermaD-ta^®^ logger TB2F, Electronic Temperature Instruments Ltd., Worthing, UK). One of the sensors was placed inside, whereas the other was placed outside of the OTC. A total of three devices were employed per population. Temperature measurements were taken at 30 min intervals during the whole experimental period. 

To assess the micro-climate conditions within individual clones, we recorded the following environmental variables inside and outside of each OTC, just before leaf sampling: air temperature (15 cm above ground), soil moisture and soil temperature (at 5 cm in depth), light intensity (photosynthetically active radiation, PAR), and Red/Far-Red ratio (RFR). Air and leaf lamina temperature were recorded with an infrared laser non-contact thermometer (Crop TRAK, Spectrum Technologies, Inc., Plainfield, IL, USA); soil moisture with a soil moisture sensor (ML3 ThetaProbe, Delta-T Devices Ltd., Cambridge, UK), soil temperature with a contact stab digital thermometer (SuperFast Thermapen, Electronic Temperature Instruments Ltd., Worthing, UK), PAR with a point quantum sensor (LI-190SA, LI-COR, Inc., Lincoln, NE, USA), and RFR with R: FR sensor (Skye SKR-110, Skye Instruments Ltd., Powys, UK).

### 4.4. Leaf Sampling and Leaf Traits Measuring

A fully developed leaf was harvested from each of the six ramets: three growing inside and other three growing outside of every OTC. Leaf sampling was performed once in each of the two growing seasons, spring and summer, during a two-year period. After sampling, the leaves were immediately packed into plastic bottles, sealed with parafilm, and transported under cold conditions to the laboratory for further analyses. 

The phenotypic values of the specific leaf area (SLA), specific leaf water content (SLWC), leaf dry matter content (LDMC), and leaf thickness (LT) were determined using standardized protocols for plant functional trait measurements [115]. Briefly, the leaf surface area was scanned as a digital image using an optical scanner (HP ScanJet 3800, Hewlett-Packard Company, Palo Alto, CA, USA; DPI) and measured using image analysis software (Image J, 1.51j8). Thereafter, the leaf samples were oven-dried to a constant mass at 60 °C for 72 h and their dry biomass was weighed immediately after cooling down. SLA (in cm^2^ g^−1^) was calculated as the projected area of a fresh leaf divided by its dry biomass. LDMC (in g g^−1^) was computed as the ratio of leaf dry to fresh biomass. SLWC (in g cm^−2^) was determined as the difference between fresh and dry leaf biomass divided by leaf area. LT was calculated according to the formula 1/SLA x LDMC [52].

### 4.5. Statistical Analyses

Because half of the experimental clones experienced manipulated air temperatures, while the other half faced ambient temperature conditions, and because we repeatedly measured several functional leaf traits on the same clonal plant at different time points (i.e., in spring and summer of two successive years), our experimental design was referred to as a repeated measures design [116,117]. According to the repeated measures terms, an individual measured at multiple time points or under different environmental conditions was named the subject; factors consisting of levels including independent groups of subjects were named the between-subjects factor, whereas time points or environmental conditions were referred to as the repeated factors or the within-subject factors [116,117]. As repeated measurements within subjects were correlated, such data require specific methods for statistical analyses [116,117].

Two approaches can be used to analyze repeated measures data for one response: analysis of variance with repeated measures (ANOVAR) and multivariate analysis with repeated measures (MANOVAR) [117,118]. In both of these analyses, the effects of interest were the between-subject effects, within-subject effects, and within-subject-by-between-subjects interaction effects. The test statistics for assessing the significance of these effects could depend on the type of effect. So, for testing the significance of the between-subject effects, the same test could be used in both univariate and multivariate analyses, while for the within-subject effects and interactions including these effects, the test statistics were different [117,118].

***Profile analysis***—To examine whether *in situ* elevated air temperatures could evoke phenotypic responses of functional leaf traits dissimilar to those induced by ambient temperatures, we implemented a multivariate statistical technique known as “profile analysis”. Profile analysis is a repeated measures extension of multivariate analysis of variance (MANOVA), which is primarily focused on profiles (vectors) of multivariate data gathered by repeated measurements of a variable from the same individual at several different points in time [117,118,119,120,121]. In this approach, the different measurements conducted on each individual should be considered as multiple dependent variables [117,119]. There were three statistical assumptions underlining the profile analysis: (1) the distribution of dependent variables is multivariate normal; (2) the variance-covariance matrix of the variables is homogenous; and (3) the dependent variables are linearly related [117,119,122].

Profile analysis served to identify whether more than one group of individuals had significantly distinct or similar profiles. Specifically, it quantified and interpreted the amount of variation related to the level and pattern effects. In this context, the mean of a vector of repeated measurements indicated the level of a profile, whereas a vector of differences between each measurement and the average profile level described the pattern of the profile [119].

***Profile plots***—To examine the relative behavior of all dependent variables from our data set, we created a profile plot for each leaf trait separately. Profile plots were generated by plotting the sample means of each dependent variable from the two groups within each of the two populations against the measured time points. One of the key objectives for generating a profile plot was to evaluate whether the profiles between the groups were parallel [119].

***Profile analysis by groups***—The multivariate data for profile analysis were obtained by repeated measurements of a leaf trait on the same clonal individuals from two independent groups that experienced different temperature conditions within their natural habitats. The profiles of the sample means for each group of plants were analyzed as lines in the profile plots. The profile analysis by the group implemented three tests that examine whether the profiles were parallel, coincidental, and flat between the groups [117,119].

Parallelism is usually the main test in profile analyses because it examines whether each segment of the profile is the same between the groups. Here, the segment was the difference in response between adjacent time points. Parallelism was tested by a one-way MANOVA, which compared multiple segments of the profile. This was a test for the interaction effect in profile analysis. An interaction occurred when the profiles were not parallel. If the null hypothesis of parallelism was rejected, there was a significant interaction between the group membership and the time points. 

Provided that the profiles were parallel, the equal levels hypothesis tested whether the profiles of the groups coincided. This test was applied to reveal whether one group, on average, had a higher level than the other over all of the time points. To do that, the grand mean of all time points was calculated for each group, and the difference between groups was tested using a univariate test. This was equivalent to the between-groups main effect in mixed ANOVA. If the null hypothesis of equal level was rejected, the group levels were significantly different from each other.

Flatness quantified the degree to which the profiles were level within any group, if parallelism was not rejected. The flatness hypothesis tested whether all dependent variables produced the same mean response. This was equivalent to the within-subjects main effect in repeated measures ANOVA. The null hypothesis of flatness is that the grand mean (averaged over treatment levels) of the set of time differences was zero. This was tested by a MANOVA (multivariate *F*). If the flatness hypothesis was rejected, there were differences in the mean values of the variables across multiple time points.

When any of the hypotheses tested by the profile analyses were significant, they were required to be followed by contrasts.

To identify the particular time interval in which the treatment effects were different, individual ANOVAs (*F*-tests) were implemented on each of the contrasts of interest. We used profile transformation to obtain the contrast variables. Profile transformation created the contrast variables as the differences between the adjacent levels of the within-subject data [117]. We tested the significance of both the treatment—by-time interaction as well as the time effect for each contrast of the within-subject factor. In these ANOVAs, mean referred to a test for the flatness hypothesis, while treatment referred to the test of the parallelism hypothesis. 

The profile analysis was run using the SAS procedure GLM in the SAS statistical package (REPEATED/PROFILE option in the SAS GLM procedure; SAS Institute, 2011) [123].

To determine the strength of the statistical association between the pairs of leaf and abiotic variables based on their ranks, Kendall rank correlation analyses were performed (PROC CORR procedure with the KENDALL option in the SAS 9.3 software; SAS Institute, 2011) [123]. The significance level was set at *p* < 0.05 to determine the statistical significance of the Kendall’s Tau correlation coefficients.

To examine the relationship between air temperature as a predictor variable and leaf traits as an outcome variable, a robust regression analysis was employed (using the PROC ROBUSTREG procedure in SAS software; SAS Institute, 2011) [123]. A significance level of *p* < 0.05 was chosen to assess the statistical significance of the regression coefficients (b) associated with the predictor variable.

## 5. Conclusions

Because global warming is now occurring faster than at any point in recorded history, one of the research questions that is still unresolved is whether the existing level of plasticity in natural populations will be enough once the global surface temperature exceeds the historical level. To answer this question, we conducted a temperature manipulation experiment in the field using open top chambers (OTCs) as the warming mechanism. OTCs simulate global warming by passively increasing air temperature. We selected 45 large clones of *Iris pumila* within two natural populations, and deployed the OTCs on one half of each clonal individual, so that each clone simultaneously experienced both the natural and experimentally increased (~1.5 °C) air temperature. In this way, we were able to single out the impact of air temperature from other co-occurring abiotic factors on the performance of the experimental plants. We assessed the phenotypic responses to warming of four functional leaf traits: SLA, LDMC, SLWC, and LT. These leaf traits reflect various aspects of resource uptake and utilization in plants. To better understand the potential role of plasticity for population persistence in a rapidly changing climate, we described the genotype-based mean temporal norms of reaction elicited in response to temperature variation for all of the leaf traits under study. We found that the genotype-based mean temporal norms of reaction of the functional leaf traits expressed at the natural and experimentally increased air temperature were mostly parallel, but not coincidental and flat. This indicates that an increase in air temperature of about 1.5 °C could evoke significant changes in the magnitude of the phenotypic expression of the analyzed leaf trait within a single vegetative generation, in general. Conversely, the direction of phenotypic changes of the same leaf traits to ambient temperature appeared to be trait-specific. Indeed, the results of robust regression analyses showed that, over the whole experiment, the association between the mean daily temperature (logged temperature (TL)) and SLA was non-significant, between TL and LDMC it was significantly positive, while between TL and both SLWC and LT it was significantly negative. As the amount of plasticity in leaf functional traits of *I. pumila* plants from contemporary populations was molded by natural selection operating within their habitats in the past, and because the RN shape of these treats expressed upon increased air temperature was parallel with that recorded at the natural temperature, we believe that the amount of plasticity in the four functional leaf traits, SLA, LDMC, SLWC, and LT, would be sufficient to shift their phenotypic values toward the fitness optimum in novel environments in the future.

## Figures and Tables

**Table 1:** The climatic variables (mean ± SE) recorded outside and inside the OTCs, over the two seasons (spring and summer) and two successive years (2018 and 2019) in two natural populations of *Iris pumila* (Population 1 and Population 2) settled in the Deliblato Sands. Statistical significance (Student’s *t*-test) of the differences: ns—non significant, * *p* < 0.05, ** *p* < 0.01, *** *p* < 0.001, **** *p* < 0.0001.

**Climatic Variable**	**Year 1**						**Year 2**					
	**Spring**			**Summer**			**Spring**			**Summer**		
	**Outside**	**Inside**	*p*	**Outside**	**Inside**	*p*	**Outside**	**Inside**	*p*	**Outside**	**Inside**	*p*
Instantaneous air temperature T_I_ (°C)												
Population 1 Population 2	32.3 ± 0.3 31.7 ± 0.4	34.3 ± 0.3 34.1 ± 0.4	**** ****	29.4 ± 0.1 31.4 ± 0.4	29.7 ± 0.2 32.3 ± 0.4	ns ***	28.2 ± 0.2 26.7 ± 0.2	28.5 ± 0.2 27.1 ± 0.2	ns ns	32.2 ± 0.3 31.5 ± 0.3	33.5 ± 0.3 32.5 ± 0.4	**** ****
Grand mean	32.0 ± 0.4	34.2 ± 0.4	****	30.4 ± 0.3	31.0 ± 0.3	***	27.4 ± 0.2	27.8 ± 0.2	*	31.9 ± 0.3	33.0 ± 0.4	****
Logged air temperature T_L_ (°C)												
Population 1 Population 2	20.0 ± 0.1 19.9 ± 0.1	21.4 ± 0.2 21.0 ± 0.1	*** ***	26.9 ± 0.2 27.0 ± 0.2	27.7 ± 0.2 29.4 ± 0.2	**** **	23.9 ± 0.1 20.7 ± 0.2	24.7 ± 0.1 23.0 ± 0.5	**** **	26.4 ± 0.1 28.1 ± 0.2	28.5 ± 0.2 29.4 ± 0.4	*** **
Grand mean	20.0 ± 0.1	21.2 ± 0.2	***	27.0 ± 0.2	28.5 ± 0.2	***	22.3 ± 0.2	23.8 ± 0.3	***	27.2 ± 0.2	29.0 ± 0.3	***
Instantaneous soil temperature (°C)												
Population 1 Population 2	18.0 ± 0.2 20.6 ± 0.4	17.5 ± 0.2 19.3 ± 0.3	* ****	23.5 ± 0.3 24.1 ± 0.3	23.0 ± 0.2 23.7 ± 0.3	** *	13.8 ± 0.3 14.1 ± 0.4	13.6 ± 0.3 12.9 ± 0.4	ns ****	24.7 ± 0.3 24.1 ± 0.4	24.8 ± 0.3 23.8 ± 0.3	ns ns
Grand mean	19.3 ± 0.3	18.4 ± 0.3	****	23.8 ± 0.3	23.4 ± 0.3	***	14.0 ± 0.4	13.3 ± 0.4	***	24.4 ± 0.4	24.3 ± 0.3	ns
Instantaneous soil moisture (%)												
Population 1 Population 2	5.0 ± 0.1 5.3 ± 0.3	4.8 ± 0.1 5.4 ± 0.3	ns ns	9.2 ± 0.4 7.4 ± 0.3	9.2 ± 0.4 7.0 ± 0.3	ns ns	8.5 ± 0.3 8.8 ± 0.3	8.6 ± 0.3 8.5 ± 0.3	ns ns	4.6 ± 0.1 4.7 ± 0.2	4.6 ± 0.1 4.8 ± 0.2	ns ns
Grand mean	5.2 ± 0.2	5.1 ± 0.2	ns	8.3 ± 0.4	8.1 ± 0.4	ns	8.6 ± 0.3	8.6 ± 0.3	ns	4.6 ± 0.2	4.7 ± 0.2	ns

**Table 2:** Means and standard errors (SE) for the four functional leaf traits: specific leaf area (SLA, in cm^2^ g^−1^), leaf dry matter content (LDMC, in g g^−1^), specific leaf water content (SLWC, in g cm^−2^), and leaf thickness (LT), in *Iris pumila* clonal individuals from two natural populations (Population 1, n = 24 and Population 2, n = 21) during the spring and summer of 2018 (Year 1) and 2019 (Year 2), expressed outside and inside of the OTCs in the Deliblato Sands.

Leaf Trait	Year 1									Year 2								
	Spring									Spring					Summer			
	Outside		Inside		Outside		Inside			Outside		Inside			Outside		Inside	
	Mean	SE	Mean	SE	Mean	SE	Mean	SE		Mean	SE	Mean	SE		Mean	SE	Mean	SE
Population 1																		
SLA	164.97	2.65	177.78	2.36	151.03	1.85	157.75	1.84		144.12	1.79	155.52	2.43		157.56	2.46	178.83	2.49
LDMC	0.156	0.001	0.151	0.002	0.223	0.003	0.222	0.002		0.205	0.002	0.202	0.002		0.193	0.003	0.183	0.003
SLWC	0.033	0.001	0.032	0.001	0.024	0.001	0.022	0.001		0.027	0.001	0.026	0.001		0.026	0.001	0.026	0.001
LT	0.039	0.001	0.037	0.001	0.030	0.001	0.028	0.001		0.034	0.001	0.032	0.001		0.032	0.001	0.031	0.001
Population 2																		
SLA	176.30	4.22	186.06	2.70	152.17	3.30	163.25	3.14		144.60	2.62	160.23	2.79		166.87	3.19	168.60	3.58
LDMC	0.157	0.002	0.157	0.002	0.231	0.003	0.228	0.002		0.195	0.002	0.190	0.003		0.191	0.003	0.186	0.003
SLWC	0.030	0.001	0.029	0.001	0.022	0.001	0.021	0.001		0.029	0.001	0.026	0.001		0.026	0.001	0.025	0.001
LT	0.036	0.001	0.034	0.002	0.028	0.001	0.027	0.001		0.036	0.001	0.032	0.001		0.032	0.001	0.031	0.001
Pooled Populations																		
SLA	170.26	2.54	181.64	2.14	151.56	1.81	160.32	1.79		144.34	1.53	157.72	1.85		161.92	2.08	174.06	2.25
LDMC	0.157	0.001	0.154	0.001	0.227	0.002	0.225	0.002		0.200	0.002	0.196	0.002		0.192	0.002	0.184	0.002
SLWC	0.032	0.001	0.030	0.001	0.023	0.001	0.022	0.001		0.028	0.001	0.026	0.001		0.026	0.001	0.026	0.001
LT	0.037	0.001	0.036	0.001	0.029	0.001	0.028	0.001		0.035	0.002	0.032	0.001		0.032	0.001	0.031	0.001

**Table 3:** Results of a repeated-measures analysis of variance (profile analysis) on the four functional leaf traits of specific leaf area (SLA, in cm^2^ g^−1^), leaf dry matter content (LDMC, in g g^−1^), specific leaf water content (SLWC, in g cm^−2^), and leaf thickness (LT) in *Iris pumila* clonal individuals from two natural populations over two seasons (spring and summer) and two years (2018 and 2019), expressed outside and inside the OTCs in the Deliblato Sands. Wilks’ λ is presented for the multivariate analyses because the F-test corresponding to this statistic is exact.

A. Between-Subjects		SLA				LDMC				SLWC				LT		
Source of Variation		df	*F*	*p*		df	*F*	*p*		df	*F*	*p*		df	*F*	*p*
Population (P)		1	3.73	0.0569		1	0.00	0.9539		1	7.84	0.0063		1	7.10	0.0092
Treatment (T)		1	32.70	0.0001		1	8.02	0.0058		1	12.42	0.0007		1	15.44	0.0002
P x T		1	0.78	0.3786		1	0.08	0.7819		1	0.33	0.5680		1	0.15	0.6966
Error		86				86				86				86		
**B. Within-subject**																
Source of Variation	Wilks’ λ	*F*	df	*p*	Wilks’ λ	*F*	df	*p*	Wilks’ λ	*F*	df	*p*	Wilks’ λ	*F*	df	*p*
Year (Y)	0.6749	41.43	1.86	0.0001	0.9550	4.05	1.86	0.0474	0.9999	0.00	1.86	0.9927	0.9986	0.12	1.86	0.7338
Season (S)	0.9761	2.05	1.86	0.1558	0.1180	643.01	1.86	0.0001	0.1543	471.19	1.86	0.0001	0.2034	336.86	1.86	0.0001
Y x S	0.2075	328.41	1.86	0.0001	0.0609	1326.2	1.86	0.0001	0.2506	257.17	1.86	0.0001	0.3792	140.88	1.86	0.0001
P x Y	0.9236	7.12	1.86	0.0091	0.7980	21.77	1.86	0.0001	0.6906	38.52	1.86	0.0001	0.6831	39.90	1.86	0.0001
P x S	0.9546	4.09	1.86	0.0462	0.8939	10.21	1.86	0.0020	0.9996	0.04	1.86	0.8516	0.9996	0.04	1.86	0.8508
P x Y x S	0.9919	0.70	1.86	0.4046	0.9597	3.61	1.86	0.0607	0.9562	3.94	1.86	0.0504	0.9507	4.46	1.86	0.0377
T x Y	0.9843	1.38	1.86	0.2442	0.9726	2.42	1.86	0.1234	0.9903	0.85	1.86	0.3603	0.9910	0.78	1.86	0.3785
T x S	0.9900	0.87	1.86	0.3538	0.9972	0.24	1.86	0.6266	0.9812	1.64	1.86	0.2031	0.9585	3.73	1.86	0.0569
T x Y x S	0.9999	0.01	1.86	0.9290	0.9860	1.22	1.86	0.2724	0.9947	0.46	1.86	0.5002	0.9710	2.57	1.86	0.1124

**Table 4:** Analyses of variance for each of the contrasts of the within-subject factor (seasons) for the level specific leaf area (SLA, in cm^2^ g^−1^), leaf dry matter content (LDMC, in g g^−1^), specific leaf water content (SLWC, in g cm^−2^), and leaf thickness (LT), in *Iris pumila* clonal individuals from two natural populations, expressed outside and inside the OTCs in the Deliblato Sands.

Source of Variation	**SLA**			**LDMC**			**SLWC**			**LT**		
	df	*F*	*p*	df	*F*	*p*	df	*F*	*p*	df	*F*	*p*
***Year1***												
*Contrast variable: summer—spring*												
Mean	1	162.03	0.0001	1	2509.31	0.0001	1	718.92	0.0001	1	448.63	0.0001
Treatment	1	0.70	0.4054	1	0.27	0.6045	1	0.13	0.7195	1	0.08	0.7784
Error	88			88			88			88		
***Year2***												
*Contrast variable: summer—spring*												
Mean	1	100.70	0.0001	1	26.17	0.0001	1	234.75	0.0001	1	27.43	0.0001
Treatment	1	0.13	0.7180	1	0.99	0.3225	1	2.04	0.1569	1	5.93	0.0169
Error	88			88			88			88		

## Data Availability

The data presented in this study are available upon request from the corresponding author. The data are not publicly available because the authors will use it in future research.
